# Pathogen-mediated selection and phylogeny shape MHC architecture in 33 bat genomes

**DOI:** 10.3389/fimmu.2026.1913384

**Published:** 2026-10-15

**Authors:** Luke W. Silver, Dominik W. Melville, Andrea L. Schraven, Magdalena Meyer, Bismark A. Opoku, Katherine Belov, Simone Sommer, Carolyn J. Hogg

**Affiliations:** 1 School of Life and Environmental Sciences, The University of Sydney, Camperdown, NSW, Australia; 2 Australian Research Council (ARC) Centre of Excellence for Innovations in Peptide and Protein Science, The University of Sydney, Camperdown, NSW, Australia; 3 Institute of Evolutionary Ecology and Conservation Genomics, Ulm University, Ulm, BW, Germany

**Keywords:** chiroptera, comparative genomics, host-pathogen co-evolution, immunity, major histocompatibility complex

## Abstract

**Background:**

Adaptive immunity is an ancestral hallmark of jawed vertebrates, yet its genomic organisation is far from static—continually reconfigured through extensive gene family duplication and evolutionary turnover. The major histocompatibility complex (MHC) plays a central role in presenting antigens derived from intra- and extracellular pathogens, eliciting pathogen-specific immune responses. Its polymorphic nature makes the MHC particularly difficult to study with genomic architecture deciphered only in a handful of species.

**Methods:**

We manually annotated 2,095 MHC-I, -II and flanking genes across 33 high quality bat genomes, covering all 21 Chiropteran families, mapping the pan-bat genomic organisation of the MHC for the first time.

**Results:**

Selection and phylogenetic conservatism drive the organization of class I and II loci, while copy number variation appears largely independent of host, ecological, or environmental factors. However, we observe suggestive trends indicating that roosting behaviour, body size, and geographic distribution may predict copy number variation at certain loci. Moreover, core gut bacterial similarity increased with structural similarity in class II loci, suggesting that bacterial communities may exert selective pressures that shapes the class II repertoires. In contrast, neither viral diversity nor similarity – estimated from a public virus database – appeared to influence class I organisation. Ancestral reconstructions indicate that similarities were present in the ancestor of both Yinpterochiroptera and Yangochiroptera species, and family-level differences emerged later, particularly in speciose families such as *Rhinolophidae, Hipposideridae* and *Vespertilionidae*.

**Conclusion:**

Overall, the genomic organization of the complete MHC region provides insight into how bat ecology may have shaped its evolution.

## Introduction

The evolution of adaptive immunity is central to the success of jawed vertebrates (1–3). The increasing availability of reference quality genomes from diverse vertebrate taxa allows evolutionary biologists to track the evolution of adaptive immunity along the tree of life (4–8). As key components of the adaptive immune system, classical major histocompatibility complex (MHC) genes have evolved to present antigenic peptides, thereby eliciting a T-cell–mediated immune response (9, 10). Hence, MHC molecules play a pivotal role in the recognition of and defence against intracellular (MHC-I) and extracellular (MHC-II) pathogens by the adaptive arm of the vertebrate immune system (11–13). MHC genes are thought to have evolved through a model of birth and death, where pathogen-mediated selection drives the duplication, deletion or pseudogenisation of loci, resulting in high levels of species-specific diversity and little orthology between species (14, 15). Owing to its extreme polymorphism, pervasive structural variation, and extensive linkage disequilibrium, the MHC genomic region has only been mapped in few animals and humans to date (8, 16–19).

Bats are the only mammals capable of powered flight (20, 21) and recognised by their enormous taxonomic and ecological diversity, as well as their unique immunity (22–24). One peculiar feature is that infected bats rarely show signs of disease (but see exceptions: (25, 26)), and it is presumed that innate and adaptive immune cascades are responsible for their high tolerance or rapid clearance of pathogens (6, 27–29). Most bat species are gregarious, living socially in colonies that either form seasonally or persist year-round (30). Their ecology and behaviour favour the exchange of viruses, fungi, parasites, and bacteria (31–35), contributing to the high diversity of pathogens they harbour (36, 37). Because bats are reservoirs for a variety of viruses with zoonotic potential, understanding MHC-I-mediated antiviral defences has taken precedence: Early work on *Pteropus alecto* - a reservoir for the Hendra virus (*Henipavirus hendraense*) - characterised the bat MHC-I region as highly condensed with only one of the three usually conserved class I duplication blocks present (38). While this analysis was conducted using a highly fragmented genome assembly sequenced with Illumina short-read technology and the investigation of the classical class I genes relied on sequencing of BAC clones, the authors suggested that the α and κ duplication block evolved stepwise from the β duplication block in derived mammals (38). Additionally, *P. alecto*’s MHC-II region is atypical in that one of twelve loci (DRB2) was located ~355 kb upstream of SYNGAP1, which delineates the end of the extended class II sub-region in other mammals (39). With the exception of DRB2, Ng, Tachedjian (39) determined that *P. alecto*’s classical DR and DQ genes as well as the non-classical DO and DM were largely comparable to other mammals, even though the classical DP genes were found to be pseudogenised. To date, there has been little other work on the genomic architecture of MHC in bats, let alone in a comparative context.

In a monumental collaborative effort, bat researchers around the globe have collated and annotated high-quality genomes from at least one representative of each of the 21 bat families (40)(*Bat1K dataset*). However, the MHC remains a challenge even for state-of-the-art assembly and automated annotation software, calling for additional manual annotation of this complex immune gene family (16, 17). We aimed to utilise this resource of new high-quality bat genomes to characterise 1) the genomic structure of the entire MHC throughout the chiropteran phylogeny, and query 2) its association with host-specific, ecological and environmental traits. We manually annotated the entire MHC region (class I, class II, class III and framework genes) in 33 bat species, covering all 21 currently recognised bat families. By integrating the MHC with vertebrate trait databases (41–43), we tested whether class I and class II structure and similarity correspond to life history variation, which can be interpreted as proxies for pathogen diversity. For a subset of taxa with publicly available virus infection records (i.e., ZOVER (44)) and data related to bat-associated gut microbiota, we additionally examined whether MHC architecture is linked to the diversity of viral and bacterial communities found among species. Finally, we reconstructed the ancestral MHC architecture for the Yinpterochiropteran and Yangochiropteran suborders.

## Methods

### Bat genomic resources

The Bat 1K consortium was established to generate chromosome level reference quality genomes for all living bat species. The data release of Phase I of the project generated chromosome-level assemblies from 103 bat species representing all 21 currently recognised families of bat (40) (*Bat1K dataset*). Reference assemblies were generated with PacBio CLR, 10x Genomics linked-read and Bionano optical maps, or PacBio HiFi or Oxford nanopore long read and 10x Genomics linked-read, with all assemblies scaffolded using HiC data. All genomes were annotated using TOGA (Tool to infer Orthologs from Genome Alignments) (45). For our analysis we selected at least one representative from each bat family with additional species included from some highly speciose families (**Supplementary Table S1**). We also included the ghost bat (*Macroderma gigas*), as no Australian bat species were included in Phase I of Bat1K, this assembly was generated using PacBio HiFi long read data and scaffolded with HiC (46).

### Manual annotation of the chiropteran MHC region

To investigate the MHC, we obtained query sequences for MHC class I, II and flanking region genes from NCBI (**Supplementary Table S2**). For each genome assembly, we used BLAST v2.11.0 (47) with the blastn algorithm to identify MHC genes. All blast hits were then manually filtered and inspected in Microsoft Excel and IGV v2.11.2 (48), respectively, to identify the highest quality blast hits and ensure each exon contained the expected splice sites, following the AG/GT convention, open reading frame as well as start and stop codons. Like MHC class I and II gene annotations, flanking genes were investigated using BLAST v2.11.0 with the blastn algorithm and default parameters (47). Blast hits for each flanking genes were filtered manually using Microsoft Excel to identify the highest quality hits. These were then subsequently checked with IGV v2.19.5 to identify open reading frames, accurate exon boundaries following AG/GT convention (48). Complete exons were extracted from each genome assembly using the getfasta function in Bedtools v2.31.0 (49). Class I and II genes were only considered complete if they contained the expected number of exons as well as complete open reading frames with no internal stop codons. Additionally, flanking genes were annotated based on both BLAST hits and evidence provided from TOGA annotations. Genomic organisation of MHC class I, II, and flanking were then visualised using the *gggenomes* package (v1.1.2; (50)) in R v4.5.1 (51).

### Ancestral state reconstruction

We visualised the number of MHC genes for each loci onto a phylogenetic tree and reconstructed the ancestral copy number of MHC genes through the *contMap* function in the *phytools* package (v2.5-2; (52)).

### Genome quality

To ensure differences in the number of MHC genes were not due to the assembly quality of each reference genome, we collated quality metrics for each assembly including; scaffold N50, percentage of gaps present in the assembly using Fasta Statistics (53) and percentage of complete mammalia_obd10 BUSCO v5.8.0 (54) orthologs present on the Galaxy Australia server (55). As scaffold N50 values were all high, with some outlier values we performed log transformation to normalise the data, additionally as BUSCO completeness and gap percentage are bounded values (0 – 1) we performed logit transformation.

### Bat trait database

We obtained data for a range of bat life history and ecological traits from the COMBINE database (43), panTHERIA (42) and Guy, Ratcliffe and Mideo (41). Traits chosen were those that could be hypothesised to influence the breadth of pathogen diversity a species could be exposed to (see Table A2 of Guy, Ratcliffe and Mideo (41) for justification). Traits that were included were mass, roost type, range size, mid-range latitude, human population density across the species range and habitat breadth. We averaged traits with records in multiple databases. Body mass, range size and human population density were log transformed owing to their skewed distribution. We analysed the absolute value of mid-range latitude to test if overall distance from the equator has an impact on MHC copy number. To compare core virus diversity and dissimilarity among the 33 bat species, we gathered data from the ZOVER database (44), including only metagenomic studies (as justified in Morales, Dong (6)). We define core virus diversity as the number of viral families that were identified in the bat family from at least two separate records. Additionally, we calculated species-by-species, mean and median core virus similarity as Jaccard distance. We proceeded in a similar way to gather core bacterial diversity and similarity, however, no database for gut bacteria exists for bats. Hence, we performed an extensive literature review of publications reporting 16S rRNA based gut microbial compositions using Google Scholar with the search terms “bat”, “gut microbiome” or “gut microbiota” (January 2026). We only included articles where the composition at bacterial family level was clearly reported or extractable from compositional plots (see **Supplementary Table S3** for a list of studies). Owing to the high variability in gut microbial composition (56), bacterial families were only considered part of the core community if they made up at least 5% of the community and were reported in at least two separate occasions in the bat family, following the same procedure as applied to viruses. Besides the number of core bacterial families, we calculated species-by-species, mean and median core bacterial similarity as Jaccard distance.

### Statistics

All statistical analyses were performed in R Statistical Software v4.5.1 (51). We performed all statistical analysis on counts of 1) each class II MHC locus 2) total numbers of class I and class II MHC and 3) all MHC loci with analyses based on Minias, Pikus (15) and Silver, Hogg and Belov (17). Firstly, we identified the most appropriate evolutionary model for each dataset using the *fitContinuous* function as part of the *geiger v2.0.11* package (57). We tested three evolutionary models: Brownian motion (BM), which is the simplest evolutionary model where a trait is assumed to evolve under genetic drift (58); BM with a scaling parameter (λ), where λ ranges from zero to one with a value of one being equivalent to BM and a value of zero indicating all species are equally related for that trait (59); and finally Ornstein-Uhlenbeck (OU), where a trait evolves to an optimum and this suggests selection (60, 61).

To determine which traits influence MHC diversity, we ran several phylogenetic least squares analyses (PGLS) using the *gls* function in the *nlme* package (*v3.1-168;* (62)). Firstly, PGLS, with a Benjamini-Hochberg (BH) FDR correction to account for multiple testing, were used to understand if the number of genes at each MHC loci or within each MHC region was influenced by the quality of genome assembly, since genome quality can influence the ability to reliably annotate complex immune regions (16). Second, bat life-history traits were analysed by PGLS in an information criterion framework, whereby a global *gls* model for each MHC locus was generated containing log(mass), roost type, habitat breadth, log(range size), abs(mid-range latitude) and log(human population density across the species range), from which all model combinations were obtained with the *dredge* function in the *MuMIn* package (v1.48.11; (63)). Following this, model averaging was performed across models within 2 AIC_c_ of the top model. For each PGLS the underlying evolutionary model was chosen as model best describing evolution at the respective MHC loci or region (i.e., the evolutionary model with the lowest AIC_c_). In cases where two evolutionary models were within 2 AIC_c_, we ran models using both competing evolutionary models. Finally, as viral or gut microbiome data were only available for a subset of species (viral N = 27; bacterial N = 25), we analysed these in a PGLS framework with both core viral/bacterial diversity mean viral/bacterial diversity similarity, with a BH FDR correction to account for multiple testing.

Unlike the MHC class I region, which formed a tightly linked genomic cluster, and hence, solely analysed in PGLS treating copy number as univariate trait evolving under alternative evolutionary models, the multi-locus architecture of the MHC-II also allowed to correlate MHC-II similarity with viral and bacterial community similarity using a mantel and partial-mantel test, the latter accounting for host phylogeny using the *vegan* package (v.2.7-3; (64, 65)).

## Results

### Manual annotation and organisation of the Chiropteran MHC

We successfully annotated the MHC class I, II, and flanking genes across 33 bat species representing all 21 currently recognised bat families. We annotated a total of 438 class I genes, 328 class II genes, and 1,329 flanking genes (**Table 1**, **Supplementary Table S4** and extended data).

**Table 1:** Summary of the number of MHC genes annotated for each MHC locus.

Species	Suborder	Family	Genus	DMA	DMB	DOA	DOB	DPA	DPB	DQA	DQB	DRA	DRB	MHCI	Flanking
*Rhinolophus affinis*	Yinpterochiroptera	Rhinolophidae	*Rhinolophus*	1	1	1	1	0	0	2	1	1	2	24	40
*Triaenops persicus*	Yinpterochiroptera	Rhinonycteridae	*Triaenops*	1	1	1	1	0	0	1	0	2	2	10	41
*Doryrhina cyclops*	Yinpterochiroptera	Hipposideridae	*Doryrhina*	1	1	1	1	0	0	2	1	1	1	23	42
*Aselliscus stoliczkanus*	Yinpterochiroptera	Hipposideridae	*Aselliscus*	1	1	1	1	0	0	2	1	1	1	13	41
*Hipposideros caffer*	Yinpterochiroptera	Hipposideridae	*Hipposideros*	1	1	1	1	0	0	3	2	1	1	18	41
*Hipposideros abae*	Yinpterochiroptera	Hipposideridae	*Hipposideros*	1	1	1	1	0	0	3	1	1	1	20	41
*Hipposideros jonesi*	Yinpterochiroptera	Hipposideridae	*Hipposideros*	1	1	1	1	0	0	2	1	3	3	16	41
*Hipposideros larvatus*	Yinpterochiroptera	Hipposideridae	*Hipposideros*	1	1	1	1	0	0	6	3	1	1	17	41
*Hipposideros swinhoei*	Yinpterochiroptera	Hipposideridae	*Hipposideros*	1	1	1	1	0	0	4	2	2	2	11	43
*Hipposideros armiger*	Yinpterochiroptera	Hipposideridae	*Hipposideros*	1	1	1	1	0	0	3	2	2	2	10	42
*Rhinopoma microphyllum*	Yinpterochiroptera	Rhinopomatidae	*Rhinopoma*	1	1	1	1	0	0	1	1	1	1	10	44
*Craseonycteris thonglongyai*	Yinpterochiroptera	Craseonycteridae	*Craseonycteris*	1	1	1	1	0	0	1	0	1	2	4	40
*Macroderma gigas*	Yinpterochiroptera	Megadermatidae	*Macroderma*	1	1	1	1	0	0	1	1	1	1	3	31
*Eonycteris spelaea*	Yinpterochiroptera	Pteropodidae	*Eonycteris*	1	1	1	1	0	0	2	1	1	1	17	40
*Rousettus aegyptiacus*	Yinpterochiroptera	Pteropodidae	*Rousettus*	1	1	1	1	0	0	2	1	1	1	3	39
*Desmodus rotundus*	Yangochiroptera	Phyllostomidae	*Desmodus*	1	1	0	0	0	0	1	1	1	1	12	39
*Mormoops megalophylla*	Yangochiroptera	Mormoopidae	*Mormoops*	1	1	0	0	0	0	1	1	1	1	13	42
*Thyroptera tricolor*	Yangochiroptera	Thyropteridae	*Thyroptera*	1	1	2	1	0	0	2	2	0	0	8	40
*Noctilio leporinus*	Yangochiroptera	Noctilionidae	*Noctilio*	1	1	0	0	0	0	1	1	1	1	5	40
*Furipterus horrens*	Yangochiroptera	Furipteridae	*Furipterus*	1	1	0	0	0	0	1	1	1	2	6	41
*Mystacina tuberculata*	Yangochiroptera	Mystacinidae	*Mystacina*	1	1	1	1	0	0	1	1	1	0	14	41
*Saccopteryx bilineata*	Yangochiroptera	Emballonuridae	*Saccopteryx*	1	1	1	1	0	0	4	3	0	7	29	41
*Taphozous melanopogon*	Yangochiroptera	Emballonuridae	*Taphozous*	1	1	0	0	0	0	3	2	1	1	11	43
*Nycteris thebaica*	Yangochiroptera	Nycteridae	*Nycteris*	1	1	0	0	0	0	4	4	1	3	16	39
*Myzopoda aurita*	Yangochiroptera	Myzopodidae	*Myzopoda*	1	1	1	1	0	0	1	1	1	0	9	39
*Natalus tumidirostris*	Yangochiroptera	Natalidae	*Natalus*	1	1	1	1	0	0	2	1	1	2	7	40
*Molossus molossus*	Yangochiroptera	Molossidae	*Molossus*	1	1	1	0	0	0	2	1	1	1	4	41
*Miniopterus schreibersii*	Yangochiroptera	Miniopteridae	*Miniopterus*	1	1	0	0	0	0	1	1	1	4	11	41
*Cistugo seabrae*	Yangochiroptera	Cistugidae	*Cistugo*	1	1	0	0	0	0	1	1	1	1	13	40
*Pipistrellus kuhlii*	Yangochiroptera	Vespertilionidae	*Pipistrellus*	1	1	0	0	0	0	1	1	1	4	6	38
*Nyctalus aviator*	Yangochiroptera	Vespertilionidae	*Nyctalus*	1	1	0	0	0	0	1	1	0	1	13	37
*Myotis myotis*	Yangochiroptera	Vespertilionidae	*Myotis*	1	1	0	0	0	0	4	2	1	2	10	40
*Myotis lucifugus*	Yangochiroptera	Vespertilionidae	*Myotis*	1	1	0	0	0	0	6	4	1	13	52	40

### MHC organisation and ancestral reconstruction

The number of genes and gene duplications varied considerably across bat species (**Table 1**). Overall, MHC class I gene copy ranged from three, identified in both *Macroderma gigas* and *Rousettus aegyptiacus*, to 52 in *Myotis lucifugus*. This substantial variation was observed among all bat species, indicating extensive expansion and contractions of class I genes within the lineage. Variation in MHC class II genes was also evident. Multiple species exhibited duplication of classical MHC class II genes, while partial deletions were observed within the DQ and DR loci in a small number of species. In contrast, non-classical DM loci were conserved, with single copies identified across all bat species analysed. Non-classical DO were consistently identified as single copies across Yingpterochiroptera species but were only present in a subset of Yangochirptera species. We also found a duplication of the DOA locus in *Thyroptera tricolor*. No classical MHC class II DP loci were detected in any bat species, suggesting deletion of this locus in Chiropteran species. Flanking genes showed variable conservation across species, with differences in both gene presence and copy number (**Table 1**). The number of flanking genes identified ranged from 31 in *Macroderma gigas* to 44 in *Rhinopomoa microphyllum*. Single gene copies predominated, with only three species exhibiting duplications of individual flanking genes and no duplicated gene shared across those species (**Supplementary Table S4**).

Across species, genes were variably distributed across scaffolds. All identified genes were located on individual scaffolds for some assemblies, forming continuous genomics regions, while in others, gene organisation was fragmented as multiple scaffolds (**Figure 1**, **Supplementary Figure S1**, **Supplementary Table S4**). Within these regions, MHC class I genes were predominately arranged as clusters with some loci more interspersed with flanking genes. The relative positioning of class I with respect to class II genes differed among species where some assemblies showed close proximity of class I and class II and flanking genes, while others exhibited separation of class I and II genes by extended regions of flanking genes or scaffold boundaries. Classical MHC-II DQ and DR loci appeared closely linked for most species (**Figure 1**). In contrast, non-classical MHC-II DM and DO loci were interspersed among flanking genes in some species and closely linked in others. Flanking genes bordered MHC regions to differing extents and contributed to variation in overall continuity we observed in MHC genomic organisation across bat species (**Figure 1**).

**Figure 1:**
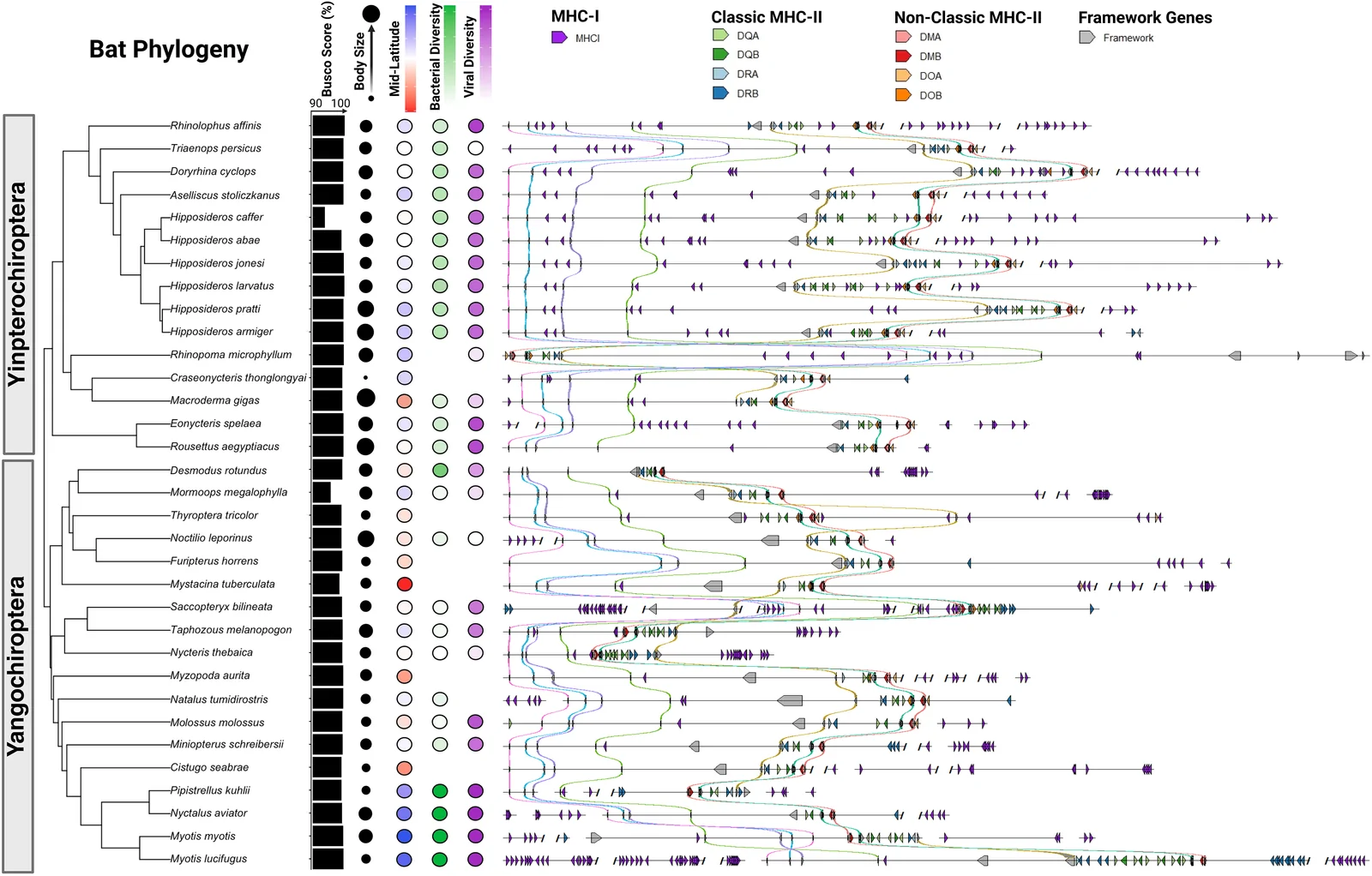
Bat phylogeny, key traits and MHC genomic organisation for 33 bat species representing all 21 currently recognised chiropteran families. The phylogeny represents a subset of the complete phylogeny constructed for 103 bat genomes and BUSCO completeness scores (90-100%) are derived from Morales, Liang (40) (*Bat1K dataset).* Body size estimate represents forearm length (mm) and are shown as scaled circle diameter. The latitudinal midpoint of each species’ distribution is shown from North (blue) to South (red) with midpoints close to the equator given as white. Core bacterial (green) and viral (purple) diversity are indicated by point fills with different intensities. The genomic organisation of MHC class I, II, and flanking genes are shown across all scaffolds where present. Lines represent scaffolds, with sections of the same scaffold represented by break lines. Arrows indicate gene orientation (forward or reverse), and the colour of the arrows represent the locus (MHC-I – purple; classical MHC-II DQA – light green, DQB – dark green, DRA – light blue, and DRB – dark blue; non-classical MHC-II DMA – light red, DMB – dark red, DOA – light orange, and DOB – dark orange; flanking genes – grey). Synteny across species is shown by conserved flanking genes; BRD2 (pink), BTNL2 (blue), POU5F1 (purple), TAP1 (green), TRIM26 (yellow), TRIM39 (aqua) and ZFP57 (red).

As with extant bat species, reconstructions of the predicted ancestral bat MHC region showed variable levels of conservation across families. For example, there is predicted to be at least three events of the deletion of the DO locus in the ancestor of the *Phyllostomidae* and *Mormoopidae*, *Noctilionidae* and *Furipteridae*, and *Vespertilionidae*, *Cistugidae*, *Miniopteridae* and *Molossidae* (**Supplementary Figure S2**). In contrast across the DR locus, there is much more conservation with ancestors of both the Yinpterochiroptera and Yangochiroptera converging to a single copy (**Figure 2**). Similarly, within the class I genes, both the Yinpterochiroptera and Yangochiroptera ancestor converge to a moderate number of genes. There are, however, some lineage specific expansions in the number of class I loci, namely *Vespertilionidae*, *Hipposideridae* and *Rhinolophidae* (**Figure 3**).

**Figure 2:**
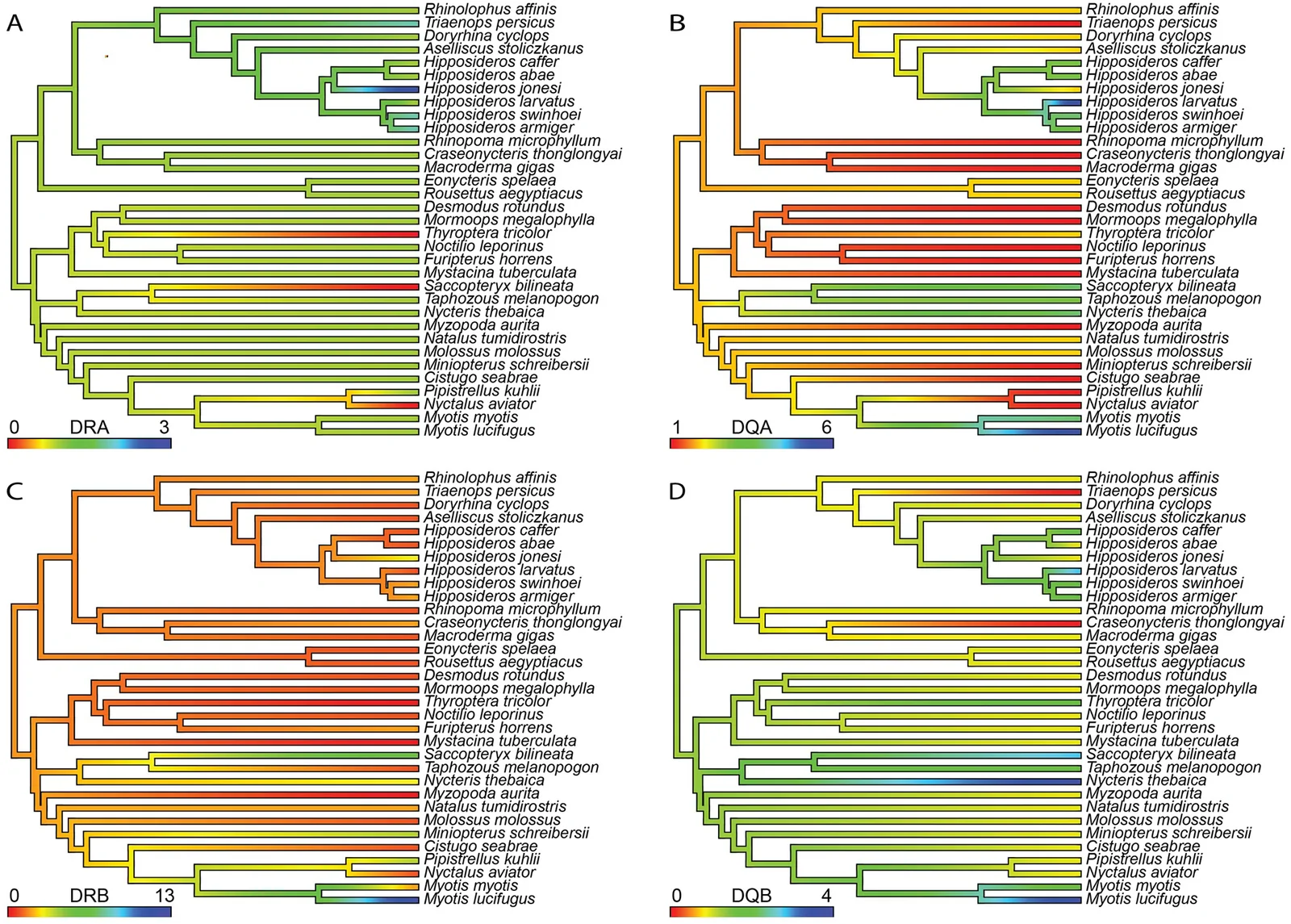
Visualisation of the copy number of classical MHC class II loci (**A**: DRA, **B**: DQA, **C**: DRB, **D**: DQB) in extant species, and prediction of the copy number in ancestral bat species generated with *contMap*, phylogeny based off Morales, Liang (40) *(Bat1K dataset)*.

**Figure 3:**
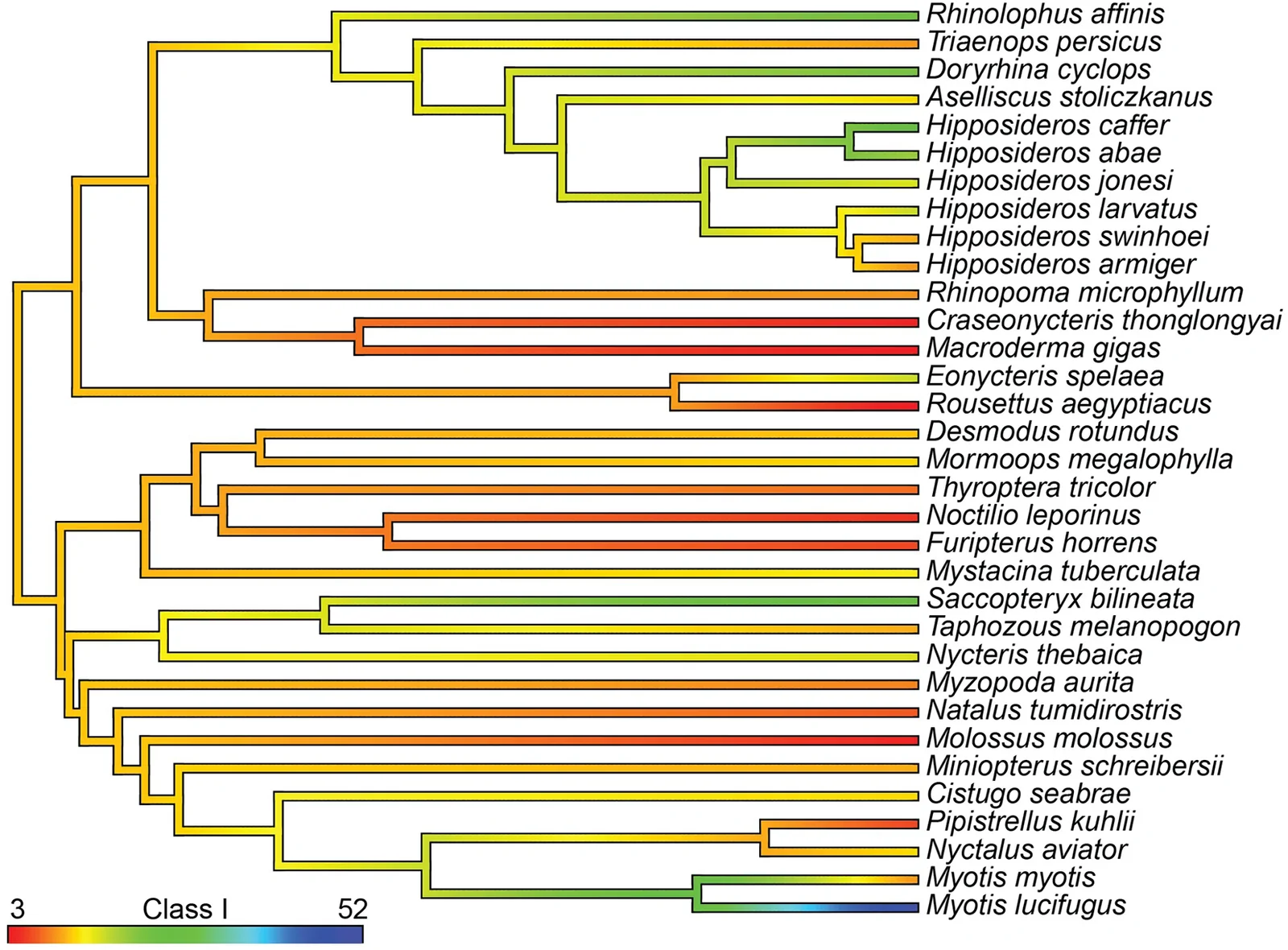
Visualisation of the copy number of MHC class I loci in extant species, and prediction of the copy number in ancestral bat species generated with *contMap*, phylogeny based off Morales, Liang (40) (*Bat1K dataset)*.

### Evolutionary models of selection

For all MHC genes, except for the non-classical DO loci, OU and BM + λ performed better than neutral Brownian motion models, indicating strong deviation from neutral evolution (**Table 2**).

**Table 2:** Selection of best evolutionary model for each MHC locus.

Gene	Evolution Model	AIC_C_	ΔAIC_C_	Relative Importance ( $\omega_{i}$)
DOA	Brownian Motion adjusted for λ	37.433	2.428	0.186
	**Brownian Motion**	**35.005**	**0.000**	**0.627**
	Ornstein-Uhlenbeck	37.431	2.425	0.187
DOB	Brownian Motion adjusted for λ	22.922	2.428	0.186
	**Brownian Motion**	**20.494**	**0.000**	**0.626**
	Ornstein-Uhlenbeck	22.892	2.398	0.189
DQA	*Brownian Motion adjusted for λ*	*119.804*	*0.535*	*0.428*
	Brownian Motion	126.964	7.696	0.012
	**Ornstein-Uhlenbeck**	**119.268**	**0.000**	**0.560**
DQB	*Brownian Motion adjusted for λ*	*93.869*	*0.362*	*0.412*
	Brownian Motion	96.843	3.336	0.093
	**Ornstein-Uhlenbeck**	**93.508**	**0.000**	**0.494**
DRA	**Brownian Motion adjusted for λ**	**58.273**	**0.000**	**0.907**
	Brownian Motion	76.641	18.368	0.000
	Ornstein-Uhlenbeck	62.822	4.548	0.093
DRB	**Brownian Motion adjusted for λ**	**157.112**	**0.000**	**0.593**
	Brownian Motion	166.013	8.901	0.007
	*Ornstein-Uhlenbeck*	*157.896*	*0.784*	*0.400*
MHCII	Brownian Motion adjusted for λ	192.201	2.783	0.189
	Brownian Motion	194.771	5.354	0.052
	**Ornstein-Uhlenbeck**	**189.417**	**0.000**	**0.759**
MHCI	**Brownian Motion adjusted for λ**	**248.571**	**0.000**	**0.700**
	Brownian Motion	259.691	11.120	0.003
	Ornstein-Uhlenbeck	250.282	1.711	0.297
ALL	**Brownian Motion adjusted for λ**	**268.296**	**0.000**	**0.535**
	Brownian Motion	276.780	8.484	0.008
	*Ornstein-Uhlenbeck*	*268.612*	*0.317*	*0.457*

Bold values represent the Evolution model with the highest relative importance.

### Genome quality analysis

PGLS analysis revealed no significant association between genome quality and our ability to identify complete MHC repertoires in bat species (**Supplementary Table S5**). Given all genomes had extremely few gaps (max 2.19%) and are highly contiguous (min scaffold N50 80.24 Mb), it is likely that we were able to identify all complete MHC genes within each species and any deletions or duplications are real.

### Host specific, ecological and environmental drivers of MHC diversity

Model averaging did not identify any effect of body mass, roost type, habitat breadth, range size, mid-range latitude or human density across the species range on copy number of MHC loci (all 95% CI of top models spanned zero, **Supplementary Table S6**). Despite this, top models for each MHC locus consistently included roost type, range-size as well as mass, suggesting there may be some influence of these traits on copy number of MHC genes. Mass consistently showed a negative trend with MHC copy number, suggestive that larger bats contain fewer MHC gene copies within their genome. Conversely, range size showed a positive trend with MHC copy number, indicating species with overall larger range sizes have more copies of MHC genes. Core viral diversity estimated from the public database ZOVER did not explain MHC-I diversity, as predicted (0.22 ± 0.360, padj-value=0.846; **Figure 4A**, **Supplementary Table S7**). Core gut bacterial diversity also showed no association with MHC-I diversity (0.61 ± 0.345, padj-value=0.486; **Figure 4B**, **Supplementary Table S7**). Because the MHC-II’s multi-region architecture portraits partially independent evolutionary trajectory and the functional breath of antigen presentation, multivariate analyses can test whether bats with similar MHC-II harbour similar viral and bacterial communities. Indeed, bats with similar MHC-II structure housed more similar gut bacterial communities (Mantel – R: 0.241, p=0.048, **Figure 4D**). By contrast, no pattern was found for viral communities (Mantel – R: 0.06, p=0.330, **Figure 4C**), which was expected since MHC-II primarily presents antigens from extracellular pathogens.

**Figure 4:**
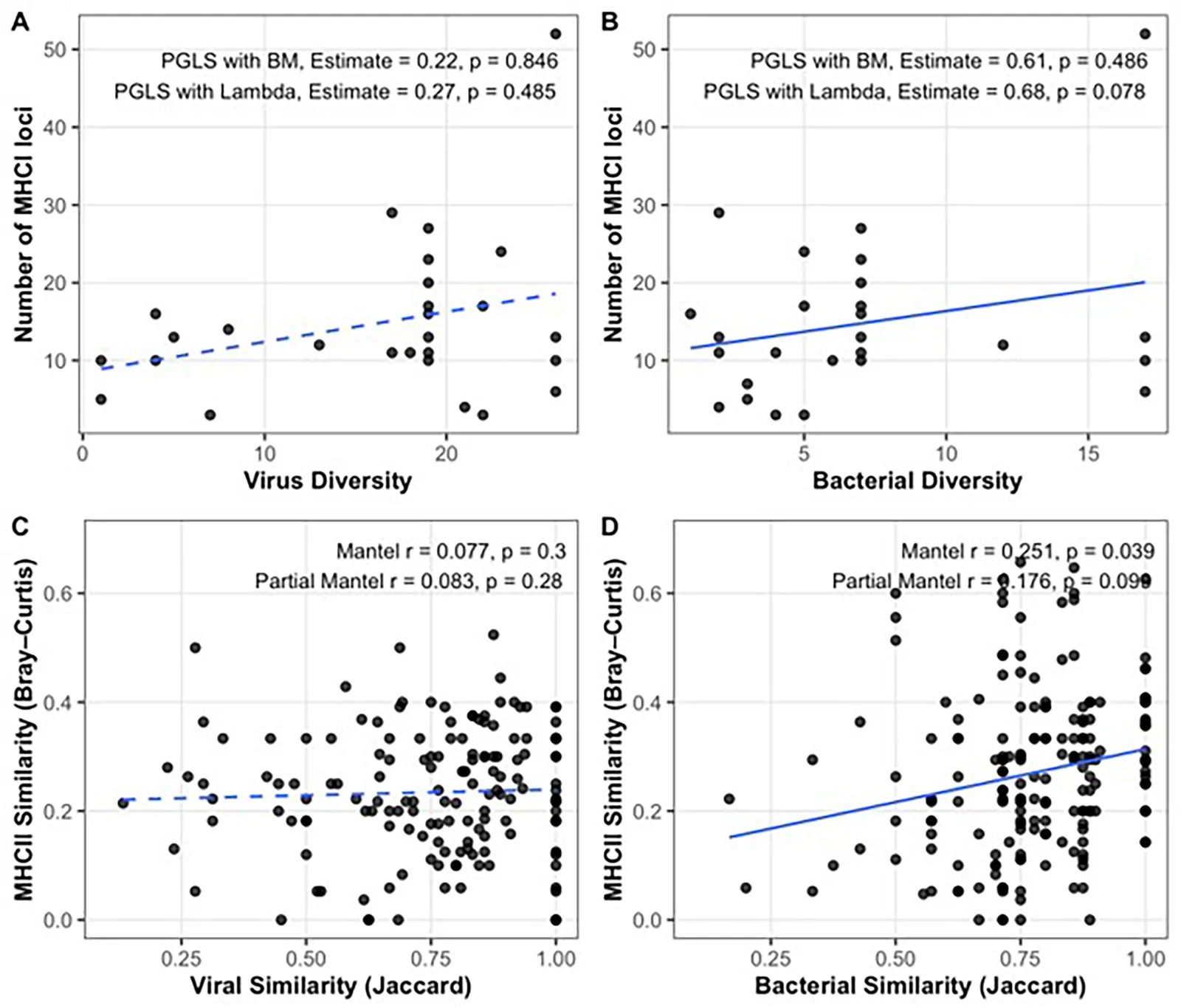
Phylogenetic least square results linking copy number variation at MHC-I with **(A)** viral and **(B)** bacterial diversity, and mantel correlations between MHC-II and **(C)** viral and **(D)** bacterial similarity. PGLS statistics given for evolutionary model considering BM and lambda, and mantel statistics given for a plain mantel test and a partial mantel test correcting for the underlying phylogenetic structure. Dashed line indicates a non-significant correlation, whereas the solid line indicates a (marginally) significant correlation.

## Discussion

Comparative genomics provides a powerful framework for uncovering shared ecological selection pressures across evolutionary lineages (e.g. Osipova, Ko (66) and Jebb, Huang (5)). Pathogen-mediated selection, while ubiquitous, varies even between closely related species and can drive both shared (i.e., trans-species polymorphism; (67)) and distinct (i.e., local adaptation, (68, 69)) evolution of host immunity. While comparative genomics has illuminated immune gene evolution in many taxa, the MHC region – known for its complexity and functional importance during pathogen peptide presentation – remains, until now, understudied in bats.

### Unique organisational features of bat MHC

Our manual annotation of 2,095 genes across 33 high-quality bat genomes revealed several unique organizational features in bat MHC regions. Notably, no DP genes were identified, contrasting earlier MHC characterisation using a highly fragmented genome of *P. alecto* (39), and DO genes were absent in some taxa, consistent with observations in other therians where certain class II loci were lost (e.g., felids and marsupials; (17, 70)). The *Myotis lucifugus* genome exhibited a particularly large number of class I genes, indicating possible lineage-specific expansions. A comparison with the MHC gene clusters characterised in *P. alecto* (38) reiterates that both conserved and divergent patterns of organisation, with gene duplication and loss events, contribute to structural variation observed in bats. Similar to Ng, Tachedjian (39), we found the MHC class II (with the exception of DOA, which is located adjacent to BRD2) genes were contained between BRD2 and BTNL2 (**Figure 1**). This MHC II organisation also reflects the typical MHC II organisation seen in humans (71). Contrasting Ng, Tachedjian (38), who suggested bat MHC class I genes were only present in the β duplication block, we found between zero (*Desmondus rotundus, Noctilio leporinus, Furipterus horrens* and *Cistugo seabrae*) and nine genes (*Eonycteris spelaea*) within this region, and two or three class I genes within the κ block in most Yinpterochiroptera. This finding mirrors the recent work of Liu, Ren (72), who identified MHC-I genes within the κ block of two *Rhinolophus* species. The largest variation in copy number occurred downstream of the class II region. We also identified class I genes interspersed within the class II genes in some species, reflecting an organisation previously observed within some marsupials (73). It has been proposed that long interspersed nuclear element sequences or retroviral elements can facilitate the reorganisation of the MHC regions (73–75). The interspersed arrangement of class I and class II genes, potentially mediated by transposable elements, illustrates how genomic rearrangements can drive structural innovation within bat MHC regions.

### Phylogenetic and pathogen-mediated selection shape bat MHC architecture

Our analyses indicated that both phylogenetic history and pathogen-mediated selection shapes the genomic architecture of classical MHC-I and -II regions in bats. As expected, classical MHC gene copy number is under evolutionary selection while, simultaneously phylogenetically constrained (15, 17, 76). In such constrained evolution the relative contributions of stabilising selection and phylogenetic covariance are difficult to disentangle. This aligned with findings in other taxa, where life history traits and ecological factors modulate immune gene evolution. For example, in felids (70), primates (77), and birds (15, 78), selection pressures were linked to reproductive strategies, migration, and immune investment. In bats, the observed high allelic diversity in a geographically widespread, migratory species suggests a capacity for adaptive immune variation (79), potentially reflecting energetic trade-offs associated with flight and the mitigation of inflammation through antioxidant mechanisms (6). Past analysis on bat genomes suggested the evolution of flight placed strong selective pressure on genes involved in DNA repair and innate immunity also inadvertently benefited their ability to resist viral infections (6, 23). This ability to tolerate oxidative stress while maintaining immune responsiveness may explain the unique patterns of MHC evolution in chiropterans. Understanding the genetic basis of these adaptations is of growing relevance in the face of climate induced range-shifts and habitat modifications that are increasing patterns of human-bat conflict and the potential for zoonotic spillover (80–82). The inherent difficulties in accurately annotating genes of the adaptive immune system (16) and lack of high quality genome assemblies for bats (5) have so far constrained our ability to fully assess how MHC evolution influences bats’ tolerance to high pathogen loads.

### MHC diversity and pathogen associations

We observed a weak but notable correlation between gut bacterial and MHC-II similarity. This is consistent with past research on bats (83), birds (84, 85) and lemurs (86), which associated individual allelic MHC variation with gut microbial diversity and fitness. In comparison to these previous studies, we found no direct relationship between viral diversity and MHC-I. This is surprising because bats are infamous for their viral diversity, and the MHC-I region is theorised as a molecular mechanism enabling tolerance to or clearance of virus infections (87, 88). While we did not find a relationship across taxa, viral pathogens may instead drive selection on within species MHC variation, for example the reduced diversity in chimpanzee (*Pan troglodytes*) MHC class I region is hypothesised to result from a selective sweep driven by simian immunodeficiency virus (SIVcpz) (77, 89). Two recent comparative analyses across Chiropteran lineages have identified signatures of selection in genes of both the innate (6) and adaptive (72) immune system, revealing extensive lineage-specific diversification in antiviral gene families, including MHC-associated pathways, that varies in gene repertoire, organisation and expression across species. These studies further show that immune gene evolution in bats is closely associated with viral exposure, with elevated viral richness linked to expanded and functionally distinct antiviral responses, alongside pervasive positive selection on pathways involved in viral detection, entry and regulation. A 3 or 5-aa insertion found among bat MHC-I is thought to have evolved to improve stability and peptide binding during elevated body temperature when flying (90). One explanation for this finding is, our statistical power was limited by the number of species for which annotated MHC and viral information is available, and the virus data is skewed towards bat species and families known to be reservoirs for zoonotic diseases (32, 35, 91). As zoonotic disease (re-)emergence and host-associated microbiota become increasingly relevant as part of the One Health framework we call for renewed efforts into standardised longitudinal virus and microbe surveillance in bats. While global efforts such as Bat1K have now given the scientific community high-quality reference genomes to answer many fundamental questions, analyses such as ours aimed to understand overarching evolutionary patterns can only be as strong as the ecological context host genetics are placed in.

### Host specific, ecological and environmental drivers of MHC diversity

As viral or bacterial diversity is laborious to assess for wildlife, life-history traits can serve as proxies for expected pathogen pressure. For example, species with broader geographical ranges or diverse foraging strategies (such as hematphagy in *D. rotundus*) are more likely to encounter a wider diversity of pathogen, with theory suggesting that a higher diversity of MHC genes will allow a population to recognise and bind antigens from more diverse pathogens (41). The widely distributed *Carollia perspicillata*, for instance, features high individual allelic MHC-I and MHC-II diversity (79, 92). We found a consistent trend for species with a larger range size having more copies of MHC genes. Immunity, however, is an energetically costly process and therefore a species must trade off energetic investment into immunity against reproduction (93, 94). Considering bats have incredibly long lifespans, typically one young per reproductive cycle, and some species feature extreme number of MHC genes, one could speculate that these species invest highly into immunity (95, 96). For instance, *Molossus molossus* has only four MHC-I genes compared to the ten in *Myotis myotis* and 52 in *M. lucifugis*, which both live nearly three times longer than *M. molossus* (97). Longer lived species may encounter a greater number and broader diversity of pathogens, necessitating more investment into immunity (41). Yet, both species also vary in the number of expressed DNA repair genes which is theorised to underpin differences in their lifespan (98). Since it is impossible to age bats based on phenotype (99), longevity data for bats is sparse. Consequently, understanding how longevity, life-history traits, and pathogen exposure shape MHC diversity in bats will require not only high-quality genomic data but also longitudinal ecological and demographic data.

### Functional insights and conservation implications

Understanding MHC genomic architecture has important conservation implications. Functional immune genes may reveal resilience in declining populations or vulnerabilities in stable or even thriving populations that neutral genomic markers may not pick up on (100–102). For instance, studies on Tasmanian devils (103) and other mammals (104) highlight the value of targeting functional loci to assess long-term viability. Bats worldwide face pressures from habitat loss, climate change and disease (105–107). Our work provides a genomic resource to enable species-specific investigations into how individual MHC allelic diversity affects susceptibility to pathogens, such as coronaviruses (108) and the white-nose syndrome causing fungus *Pseudogymnoascus destructans* (109). Using functional genomic markers can therefore inform more nuanced conservation strategies (110).

## Conclusion

This study provides the first comprehensive annotation of the MHC region across multiple bat species, revealing unique organizational features, the interplay of phylogeny and pathogen-mediated selection, and links to microbial communities. By integrating comparative genomics with ecological and life-history traits, we illuminate the evolutionary dynamics of bat immune systems and provide a framework for future studies on host-pathogen interactions and conservation genomics. Our findings underscore the need for continued virus, microbiome and ecological surveillance to contextualise the unique MHC diversity in bats.

## Data Availability

The original contributions presented in the study are included in the article/**Supplementary Material**. Further inquiries can be directed to the corresponding author.
