# Supplementary material for: Pathogen-mediated selection and phylogeny shape MHC architecture in 33 bat genomes
Source: Front Immunol. 2026 Oct 15;17:1913384. doi: 10.3389/fimmu.2026.1913384 (PMC13716760; doi:10.3389/fimmu.2026.1913384)
Supplement: Supplementary file 1 [file SupplementaryFile1.docx]

## Supplementary Figures

Figure S1: The genomic organisation of MHC class I, II, and flanking genes are shown across all scaffolds where present. Lines represent scaffolds, with sections of the same scaffold represented by break lines. Arrows indicate gene orientation (forward or reverse), and the colour of the arrows represent the locus (MHC-I – purple; classical MHC-II DQA – light green, DQB – dark green, DRA – light blue, and DRB – dark blue; non-classical MHC-II DMA – light red, DMB – dark red, DOA – light orange, and DOB – dark orange; flanking genes – grey).

Figure S2. Visualisation of the copy number of non-classical DO loci in extant species, and prediction of the copy number in ancestral bat species generated with *contMap*, phylogeny based off *Morales et al. (under review).*
